# FM19G11 and Ependymal Progenitor/Stem Cell Combinatory Treatment Enhances Neuronal Preservation and Oligodendrogenesis after Severe Spinal Cord Injury

**DOI:** 10.3390/ijms19010200

**Published:** 2018-01-09

**Authors:** Ana Alastrue-Agudo, Francisco Javier Rodriguez-Jimenez, Eric López Mocholi, Francesca De Giorgio, Slaven Erceg, Victoria Moreno-Manzano

**Affiliations:** 1 Neuronal and Tissue Regeneration Laboratory, Príncipe Felipe Research Center, 46012 Valencia, Spain; aalastrue@cipf.es (A.A.-A.); frodriguez@cipf.es (F.J.R.-J.); elopezm@cipf.es (E.L.M.); f.degiorgio85@gmail.com (F.D.G.); 2 Stem Cell Therapies in Neurodegenerative Diseases Laboratory, Centro de Investigación Príncipe Felipe, 46012 Valencia, Spain; serceg@cipf.es

**Keywords:** FM19G11, spinal cord injury, ependymal progenitor stem cells, oligodendrogenesis, locomotion, neuronal regeneration, axon growth

## Abstract

Spinal cord injury (SCI) suffers from a lack of effective therapeutic strategies. We have previously shown that individual therapeutic strategies, transplantation of ependymal stem/progenitor cells of the spinal cord after injury (epSPCi) or FM19G11 pharmacological treatment, induce moderate functional recovery after SCI. Here, the combination of treatments has been assayed for functional and histological analysis. Immediately after severe SCI, one million epSPCi were intramedullary injected, and the FM19G11 compound or dimethyl sulfoxide (DMSO) (as the vehicle control) was administrated via intrathecal catheterization. The combination of treatments, epSPCi and FM19G11, improves locomotor tasks compared to the control group, but did not significantly improve the Basso, Beattie, Bresnahan (BBB) scores for locomotor analysis in comparison with the individual treatments. However, the histological analysis of the spinal cord tissues, two months after SCI and treatments, demonstrated that when we treat the animals with both epSPCi and FM19G11, an improved environment for neuronal preservation was generated by reduction of the glial scar extension. The combinatorial treatment also contributes to enhancing the oligodendrocyte precursor cells by inducing the expression of Olig1 in vivo. These results suggest that a combination of therapies may be an exciting new therapeutic treatment for more efficient neuronal activity recovery after severe SCI.

## 1. Introduction

Traumatic spinal cord injury (SCI) is a devastating disorder with loss of neurological function immediately below the affected segment with no currently effective treatment for the subsequent paralysis. The failure to recover from SCI in adult mammals is attributed to both extrinsic and intrinsic factors [1]. The extrinsic factors include a lack of appropriate trophic support like brain-derived neurotrophic factor (BDNF), basic-fibroblastic growth factor (bFGF), vascular endothelial growth factor (VEGF) [2], stromal derived factor 1 (SDF1) [3] or insulin-like growth factor 1 (IGF-1) [4], since among others, they have shown neuroprotective effects when added ectopically.

In the primary injury phase, the direct impact causes cell death at the injury site with axonal damage leading to the interruption of the ascending and descending spinal pathways. The blockade of nerve conduction generates paralysis and temporary loss of neural functions by spinal shock. The primary injury leads to massive cell death of neurons, glial cells and endothelial cells lining the blood vessels. The surviving neurons at the lesion site respond with a barrage of action potentials that create significant local shifts in ion levels and cause also the release of excitatory neurotransmitters (i.e., glutamate), resulting in the death of more nearby neurons [5]. The inflammatory response after SCI is initiated by peripherally-derived immune cells (macrophages-monocytes, neutrophils and T-cells) and glial activation (astrocytes and microglia) [6,7,8]. Macrophages and microglia contribute to the inflammatory response through the release of cytokines like tumor necrosis factor α (TNF-α), interleukin 1 or 6 (IL1 or IL6) [9] in an initial phase; however, later, additional anti-inflammatory cytokines like IL10 are known to contribute to tissue remodeling [10,11]. The secondary phase is also marked by the massive death of oligodendrocytes. The combination of demyelination and altered ion channel function may lead to changes in surviving neurons that can produce central chronic pain. The lesion site forms a fluid-filled cavity delimited by the reactive scar acting as a containment barrier forming an impenetrable wall with extracellular matrix proteins and chondroitin sulfate limiting the axonal regrowth [12], thereby inhibiting the regenerative potential of those axons to reconnect with the distal segments [13]. SCI is a complex and multifactorial cascade of events that progressively reduces the chances of the limited endogenous regenerative capacity to rescue the lost connections. Therefore, therapeutic interventions need to rapidly interfere with this degenerative cascade via powerful neuroprotective activities, promoting in addition functional neuronal plasticity. A combination of neuroprotective and neuroregenerative treatments constitutes the current key strategies for the creation of an efficient SCI treatment. Several strategies have already shown the efficiency of the combination of cell therapy and pharmacological approaches [14]. Transplantation of neural stem/progenitor cells (NSPCs) has shown promising results in the repair and regeneration of lost neural tissues and the associated restoration of neurological deficits [14,15,16]. NSPCs include multipotent stem cells present in the periventricular subependymal layer and the subgranular zone of the dentate gyrus in the brain, as well as in the ependymal regions lining the central canal of the spinal cord (epSPCs) [13]. NSPCs represent an ideal candidate for stem cell-based SCI therapy based on noted functional improvements after transplantation and the absence of malignant transformation, offering a safe and relevant cell type for clinical applications. After SCI, epSPCs proliferate and migrate to the injured area and produce new oligodendrocyte progenitors [14]. We previously showed that acute transplantation of undifferentiated epSPCs from SCI donors (epSPCi) or in vitro differentiated OPCs into a rat model of severe spinal cord contusion produced significant locomotion recovery from one week after injury [15]. Moreover, we recently demonstrated the advantages of the synergistic activity of the polyacetal curcumin (PA-curcumin), a new polymeric conjugate of curcumin, with epSPCi in rats with chronic SCI. A single administration of the combinatory treatment into the intrathecal space significantly rescued the locomotor activity of the paralyzed rats, as opposed to ineffective individual treatments [17]. The PA-curcumin produced neuroprotective and anti-inflammatory capabilities, as previously reported [18,19,20,21,22], but also showed regenerative activity by increasing axonal elongation-related mechanisms. The epSPCi, with multipotent characteristics, lining the central canal of the spinal cord [15], after SCI, proliferate and migrate to the injured zone, giving rise to new oligodendrocyte progenitors [23]. Acute transplantation of epSPCi efficiently reversed the paralysis associated with acute SCI in rats [24,25]. The transplanted cells migrated long distances from the rostral and caudal regions to reach the neurofilament-labeled axons in and around the lesion zone, while epSPC transplanted animals always showed fewer cavities and a smaller scar area [25]. In the chronic scenario, although epSPCi improved the locomotion, only partial success was found in comparison with the acute treatment [26]. However, the combination of treatments, for instance by combining PA-curcumin and epSPCi, significantly induced both β-tubulin-III (neurofilament marker for neuronal projections) and GAP43 (axon growth marker) expression at the epicenter of the injury in comparison with the individually-treated groups [17].

FM19G11 was first identified as an inhibitor of HIFα protein expression and transcriptional activity under hypoxic conditions repressing a variety of key genes involved in stemness. Moreover, directed differentiation experiments demonstrated that FM19G11 favors oligodendrocyte differentiation blocked under hypoxia, possibly through the negative modulation of Sox2 (sex determining region Y-box 2) and Oct4 (octamer-binding transcription factor 4) expression and by allowing the epSPCs to differentiate under hypoxia [27]. However, under normoxic conditions, FM19G11 modifies the mitochondrial uncoupling process, by induction of UCP sensors, which induces glucose uptake by activation of AMPK (AMP-activated protein kinase), as well as AKT (Protein Kinase B) signaling pathways. The consequence of these molecular events is an increase of the self-renewal machinery of different stem cell populations, including the epSPCs. FM19G11 induces early glycolytic-related responses associated with PI3K/AKT/mTOR signaling induction [28,29]. Interestingly, when FM19G11 is infused during three days starting immediately after SCI, in the intrathecal space, it induces functional locomotion recovery one month after treatment, which is associated with an increase of vimentin expression and in the number of neuronal fibers in the injured area [30].

Here, we look for an enhanced effect to rescue neuronal activity for progressive motor function in severe SCI using a combination that includes cell therapy, epSPCi and the pharmacological treatment based on the local application of FM19G11.

## 2. Results

### 2.1. FM19G11 or epSPCi Improve Locomotion in Severe SCI with No Synergistic Effect

**Figure 1:**
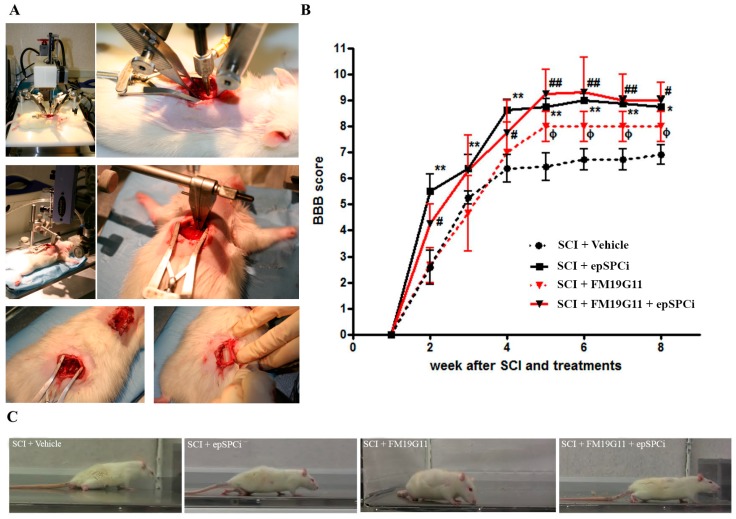
FM19G11, epSPCi or the combination of both improve locomotion in severe spinal cord injury (SCI) in comparison with the control group. (**A**) Surgery strategy to induce severe SCI by a 250-kdyn impact and acute transplantation by intramedullary injection of epSPCi and intrathecal catheterization for local and sustained delivery of FM19G11 using an osmotic pump; (**B**) functional locomotor analysis (BBB scores) over eight weeks post-treatment with vehicle, as a control, epSPCi, FM19G11 or FM19G11 + epSPCi; quantitative data are expressed as the mean ± S.E.M.; *, or #, or ф, *p* < 0.05, ** or ##, *p* < 0.01; (**C**) representative capture images from video recordings of one animal of every group for BBB analysis at the eighth week after SCI are shown as follows: SCI + vehicle (control), SCI + epSPCi, SCI + FM19G11, SCI + FM19G11 + epSPCi.

Individual treatments with FM19G11 by intrathecal administration [30] or epSPCi by intramedullary transplantation [24,25] improved locomotion when applied immediately after injury. Herein, in order to study a potential additive or synergistic effect among both treatments, a combination of both was assayed. The epSPCi, or growth medium in the control group, were transplanted by intramedullary injection at the injured area immediately after spinal cord contusion (Figure 1A, top pictures). Afterwards, the pharmacological treatment was performed by continual delivery of FM19G11 or DMSO (in the control group), using an osmotic pump, connected to a catheter, into the intrathecal space (Figure 1A, bottom pictures). All treated animals distributed into control, epSPCi, FM19G11 and FM19G11 + epSPCi groups were individually videotaped, and locomotor functional recovery was scored blind by two unbiased observers by using the 21-point Basso, Beattie, Bresnahan (BBB) scale. All three treated animals had a significant increase in the BBB scores after the fourth week, in comparison with the control group (control: 6.3 ± 0.26; epSPCi: 8.8 ± 0.4 *; FM19G11: 8 ± 0.6 *; FM19G11 + epSPCi: 9 ± 0.7 *; *, *p <* 0.05 vs. control). However, no significant differences were found among the three treatments (Figure 1B). Interestingly, epSPCi transplanted animals (epSPCi and FM19G11 + epSPCi) always showed better maximum scores in the BBB test at shorter times after SCI (Figure 1B). The frame captures of representative video tape recordings in the open field test, eight weeks after surgery, showed a typical plantar placement of the paw with weight support instance, consistently found in animals treated with the combination of treatments, as well as the weight support by dorsal stepping, consistently found in the epSPCi group. Plantar placement of the paw with no weight support in the stepping was also frequently found in the FM19G11-treated group. The control animals did not show any stepping, but occasionally sweeping, without showing any weight support (Figure 1C).

### 2.2. FM19G11 Intrathecal Administration after SCI Preserves Spinal Cord Tissue

**Figure 2:**
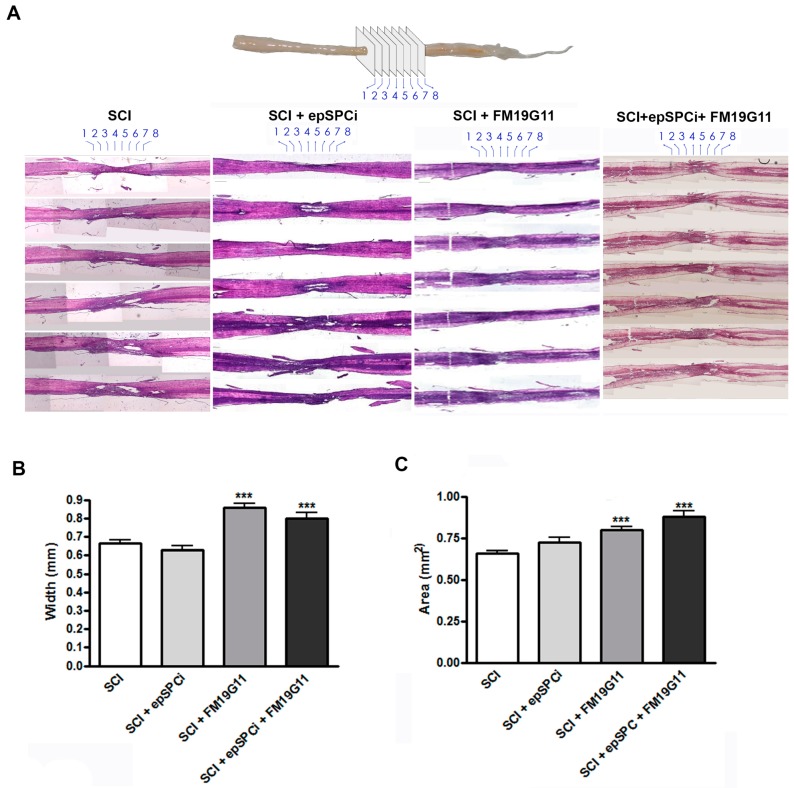
FM19G11 preserves spinal cord tissue. (**A**) Top panel: spinal cord model showing the eight selected points for the quantification of the width and the lesion area at the spinal cord injury epicenter; bottom panels: representative histological sections of hematoxylin/eosin staining for each experimental condition; Scale bar = 500 µM; (**B**) quantification of the whole width epicenter area, including all eight selected points; (**C**) quantification of the lesion area considering the elliptic shape of the spinal cord, the higher and smaller radius at each section. Quantitative data are expressed as the mean ± S.E.M; *** *p* < 0.001 in comparison with SCI (control).

The lost tissue due to the destructive secondary damage including the inflammatory reaction following the SCI was evaluated. As is indicated in the illustrative model (Figure 2A, top representation of the analyzed planes in the spinal cord), the quantification of the spinal cord width (Figure 2B) at the injury area (Figure 2C) at eight consecutive points along four mm of the epicenter of the lesion was performed. The spinal cord injury area was monitored via hematoxylin-eosin (H&E) histological analysis, eight weeks post-injury (Figure 2A, bottom panels). The comparative analysis among all the groups after quantification of the spinal cord width at the injury epicenter demonstrated that since larger tissue degeneration remained in the SCI site following control treatment or epSPCi transplantation alone, FM19G11- and FM19G11 + epSPCi-treated rats presented wider cords (Figure 2B). The quantification of the total spinal cord area at the epicenter of the injury, by taking into account the elliptic shape of the cord and including the width and the length average, showed the same result. The FM19G11 and FM19G11 + epSPCi groups presented higher total areas in comparison with the control or epSPCi groups, tending to recover a closer shape to the non-injured tissue (Figure 2C).

### 2.3. FM19G11 and epSPCi Combination Treatment Reduces the Scar Extension and Astrogliosis and Increases the Neuronal Fibers at the Injury Area

**Figure 3:**
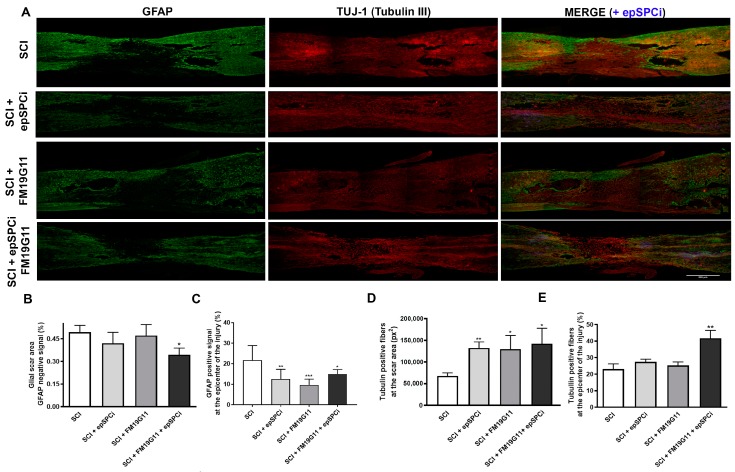
Combinatory treatment reduces the glial scar formation and preserves larger numbers of neuronal fibers. (**A**) Representative immunofluorescence images from glial fibrillary acidic protein (GFAP; **green**), β-tubulin-III (**red**) and merge including green fluorescence protein (GFP)-epSPCi in the transplanted groups (GFP, **blue**) with positive signals at the epicenter of the injury from spinal cord longitudinal sections in every experimental condition; Scale bar, 500 μm; (**B**) quantification of the negative area or (**C**) the positive signal at the injury site for GFAP expressed as a percentage of the total epicenter area, equivalent in length for all tested groups; (**D**) quantification of the positive signal for β-tubulin-III (tubulin) at the negative GFAP area or (**E**) covering all the injury area, expressed as a percentage of the total epicenter area, equivalent in length for all tested groups. Quantitative data are expressed as the mean ± S.E.M; * *p* < 0.05, ** *p* < 0.01, *** *p* <0.001 in comparison with SCI (control).

The glial scar has been accepted to be delimited by the astrocytes expressing glial fibrillary acidic protein (GFAP) [7]; then, as we previously reported [17,25,30], we evaluated the extension of the glial scar by measuring the negative GFAP stained area at the epicenter of the injury (Figure 3A,B). We found that only the combinatory treatment, FM19G11 and epSPCi, decreased the extension of the astrocytic negative area in comparison with the control group or the individual treatments (Figure 3B), indicating a reduction of the glial scar formation, creating a more permissive neuronal environment. However, all treatments, FM19G11 or epSPCi or their combination, reduced the astrogliosis based on a lower GFAP positive staining at the epicenter of the lesion, surrounding the scar (Figure 3A,C). Further assessment at the glial scar area was performed by measuring the β-tubulin-III-positive staining for neuronal filament for those neurons crossing the glial scar. All treatments, epSPCi, FM19G11 and the combination of both, were found to always show a larger positive area for β-tubulin-III in comparison with the control group (Figure 3D), indicating an independent effect of the individual treatments on neuronal fiber preservation and the glial scar formation.

However, when the β-tubulin-III positive area was quantified at the total epicenter of the injury, including the rostral and caudal tissue surrounding the scar, only the animals that received the combination of treatments, FM19G11 + epSPCi, presented a significant result in comparison with the control group (Figure 3E).

### 2.4. FM19G11 and epSPCi Induce the Expression of GAP43 Expression, an Axon Growth Marker, and RIP, a Marker for Myelinated Oligodendrocytes at the Injury

**Figure 4:**
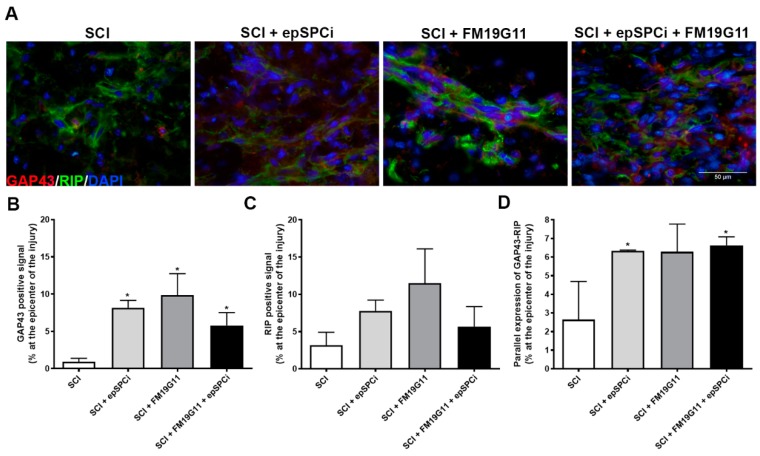
Treatment with FM19G11 plus epSPCi preserves larger numbers of neuronal fibers. (**A**) Representative immunofluorescence images at high magnification of receptor interacting protein (RIP, **green**) and GAP43 (**red**) expression at the epicenter of the injury are shown for every experimental condition (scale bar, 50 μm); (**B**) quantification of the GAP43 (growth cone marker); (**C**) RIP (mature oligodendrocyte marker) or (**D**) the parallel detection of the GAP43 and RIP-positive signal at the epicenter of the injury expressed as a percentage of the total epicenter area, equivalent in length for all tested groups. Quantitative data are expressed as the mean ± S.E.M. (ANOVA), * *p* < 0.05 in comparison with SCI (control).

GAP43, a known marker for axonal cone growth formation [31], was assayed to analyze the capacity of each treatment to favor neuronal plasticity. Encouragingly, eight weeks after injury and treatments, a significant induction of the GAP43 positive signal was detected along the neuronal fibers at the injured site by all treated groups in comparison with the non-treated animals (Figure 4A,B). The substantial increases in β-tubulin-III (Figure 3D,E) and GAP43 labeling for the combinatory treatment indeed suggest a more efficient relationship between the increased preservation in the number and quality of axon fibers. Interestingly, although no significant differences in the expression for the receptor interacting protein (RIP), a cell marker for mature oligodendrocytes by detecting 2′,3′-cyclic nucleotide 3′-phosphodiesterase [32], were detected when the control group was compared to any treatment (Figure 4C), the detected signal of GAP43 maintains a significant parallel expression with the mature and myelinated axons stained with RIP in the epSPCi transplanted groups (Figure 4D), as is also shown in the representative magnified images of double staining detection of GAP43 (red) and RIP (green) for each experimental condition (Figure 4A).

### 2.5. FM19G11 Favors Oligodendrocyte Replacement

**Figure 5:**
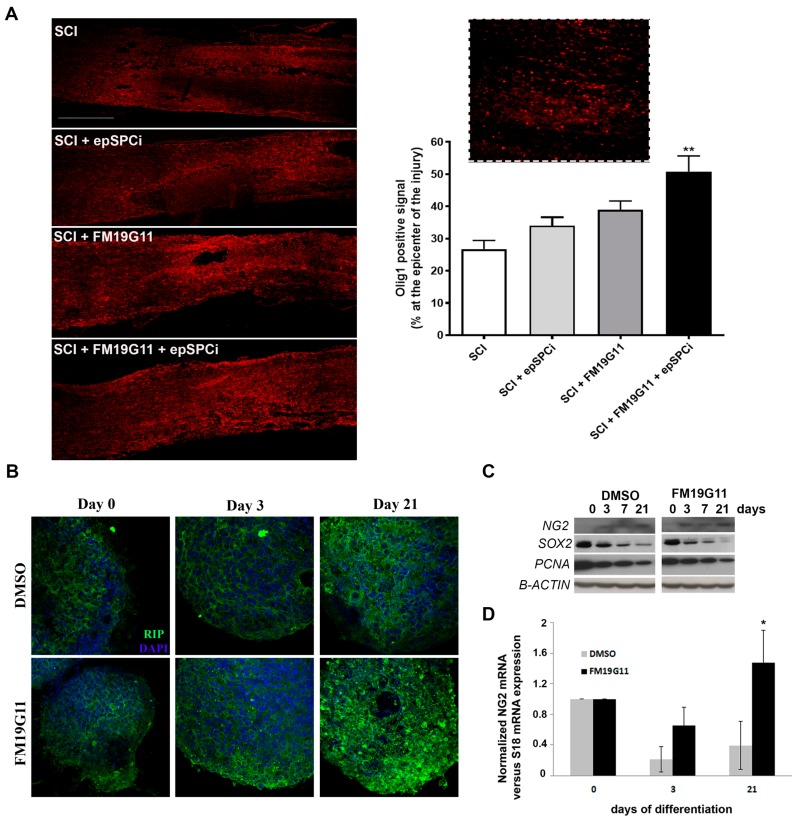
FM19G11 promotes in vivo and in vitro oligodendrocyte turnover. (**A**) (**left** panel) Representative immunofluorescent images of the Olig1-positive signal at the epicenter of the injury in every experimental condition (scale bar, 500 μm); (**right** panel) quantification of the positive signal for Olig1 expressed as a percentage of the total epicenter area, equivalent in length for all tested groups. Inset: high magnification of Olig1 immunostaining. Quantitative data are expressed as the mean ± S.E.M. ** *p* < 0.01 in comparison with SCI (control); (**B**) representative immunofluorescent images of RIP (mature oligodendrocyte marker) at different time points through the directed induced oligodendrocyte differentiation process of epSPCi in culture in the presence of FM19G11 or vehicle (DMSO) (scale bar, 500 μm); (**C**) western blotting of NG2, Sox2, PCNA and β-actin semi-quantitative expression and (**D**) quantitative RT-PCR analysis for the expression of NG2 (oligodendrocyte marker) at the different stages of the directed in vitro differentiation process into oligodendrocytes from epSPCi in the presence of FM19G11 or DMSO (vehicle), * *p* < 0.05 in comparison with DMSO.

It is well established that after SCI, which causes extensive oligodendrocyte cell death [33], surviving oligodendrocyte progenitor cells (OPC) are the major source for oligodendrocyte replacement and remyelination [34]. It also has been shown that, over the first weeks post-injury, the OPCs differentiate into mature oligodendrocytes, particularly along the lesion borders, and contribute to the re-myelination process [35,36,37]. The detection of Olig1 expression, an early OPC marker [38], eight weeks after SCI, demonstrates a significant induction of this population by the FM19G11 treatment in combination with epSPCi transplantation (Figure 5A). However, at the time of sacrifice, no significant differences were found in the RIP population (Figure 4C), indicating a lack of maturation induction of the proliferating OPC by the treatments. FM19G11 was previously shown to allow the oligodendrocyte differentiation in vitro efficiently when it was delayed under hypoxia [27]. Here, we found that the presence of FM19G11 also improves the maturation of epSPCi into oligodendrocyte precursor cells measured as an increment of RIP expression at the protein level (Figure 5B) and NG2 expression at the protein (Figure 5C) and mRNA levels (Figure 5D) with a parallel decrease of the proliferative marker PCNA (proliferating cell nuclear antigen) or the stemness factor Sox2 (Figure 5C).

## 3. Discussion

A combination of strategies with a synergistic effect of improved neuroprotection and neural plasticity may lead to significant improvements not yet achieved by unique treatments. Here, we show how the combinatory use of FM19G11 and epSPCi reduces glial scar formation, reduces astrogliosis, increases the number of neuronal fibers at the epicenter of the lesion, increases the expression markers for neuronal plasticity and induces oligodendrocyte turnover for potential re-myelination.

Traumatic contusion injury generates a rim of spared white matter tissue, with extensive neuronal degeneration and glial scar formation generating an incomplete lesion with a variable remaining demyelinated, but initially non-interrupted axons depending on the initial severity of the trauma, which, in fact, provides a more realistic experimental setting mimicking the clinical lesions to test potential neuroprotective and additional neurorestorative strategies. Extensive proliferation of different cell types occurs at certain time points, like oligodendrocyte, neural precursor cells and also astrocytes and microglia, in a more reliable way than the transection models [39]. Here, we induced a severe contusion by applying a force of 250 kdyn (kilo dyne) in rats of ~200 g, limiting the spontaneous functional regeneration during the first weeks after injury in comparison with moderate injuries and completely impairing it at the chronic stages (one month after injury), allowing the restorative functional evaluation among the different experimental groups in a shorter period than for instance in models of a complete section [40].

We recently showed that transplantation of epSPCi, the ependymal cells that represent the latent neural stem cell population in the adult spinal cord, activated and induced to proliferate in vivo after spinal cord injury [33] in combination with PA-curcumin significantly improves the locomotion in a chronic and sever traumatic SCI [17]. Here, we demonstrated the beneficial contribution to the epSPCi transplantation of the addition of FM19G11 previously reported to modulate proliferation and differentiation of this cell population and to have neuroprotective properties when applied immediately after injury [30]. When all treatments were compared, the cell transplanted groups, epSPCi and FM19G11 + epSPCi, showed faster functional rescue by measuring locomotor tasks, significantly different from the locomotor recover found in the vehicle- or the FM19G11-treated group. Neither vehicle- nor FM19G11-treated animals exhibited coordinated movement, but occasional weight support or plantar steps. The NPC progeny were found to be necessary to produce several neurotrophic factors that support neuronal survival after injury [41]. A2B5-positive glial cells have been shown to promote neuroprotection and repair of the injured spinal cord, promoting axonal sprouting when induced in vitro to differentiate into a more astroglial fate [42]. Accumulating evidence in the last decade defends the beneficial role of the astrocytes in response to SCI, giving support to the survival cells and allowing their processes [8]. We found, eight weeks after injury, a significant decrease of the GFAP-positive reactivity for astrocyte detection at the epicenter of the injury in response to all treatments, with an increase in the neurofilament-positive fibers. However, for better interconnection of both events, additional experimentation at a shorter time of analysis or specific deletion of the astrocytic population will be required to understand a potential associated mechanism of action.

The global detection of infiltrating macrophages, positive for CD68-ED1, eight weeks after injury, did not show significant differences in any of the tested conditions; however, it has been shown by others that the focal transplantation of NPCs decreases the relative proportion of classically-activated M1-like cells among the macrophage lineage cells infiltrating the injured cord [43], favoring the shift of the balance towards tissue remodelling/repair associated also with the M2-like cells and facilitating the recovery from spinal cord injury-induced secondary damage, undetected in a global analysis [44]. The lower astrogliosis activation found in all treated groups would additionally contribute to reduce the scar extension; the combined treatment exhibited in fact the best functional outcome with reduced spared tissue, where the combination with FM19G11 with an additional neuroprotective effect could contribute to favoring environmental homeostasis. It was very interesting that GAP43, previously described as a growth cone marker for growing axons, was induced in all treated animals at the scar area showing a similar effect as TUJ-1. Moreover, the growing axon ends positive for GAP43 were mostly accompanied by myelinated oligodendrocytes when the animals were treated with FM19G11, or with epSPCi, or both, supporting more effective neuronal plasticity in comparison with non-treated animals.

At the SCI area, the vascular network is lost with edema and disruption of the BSCB, extensively justifying the local application at the subarachnoid space of the pharmacological treatment. We previously showed that FM19G11 intrathecal administration immediately after SCI accelerates locomotor recovery in the rats one month after treatment. Indeed, the sustained administration of FM19G11 during one week showed a plausible neuroprotective role, preserving more neurofilament β-tubulin III-positive fibers in the injured area surrounded by an increased number of neural precursor vimentin-positive cells [30]. Here, we found that this neuroprotective effect by FM19G11 treatment was maintained, as was determined by the histological analysis, for two months. The analysis of both the in vitro and in vivo mechanism of action of FM19G11 reveals an important influence on the mitochondrial activity by early activation of UCP proteins, and therefore, the compound modifies oxidative metabolism at least in the epSPCi population by activation of the AKT/mTOR signaling pathway, which creates an adaptive response in the cells by increasing the GLUT-4 receptors [29,30] and probably improving the exocytosis and exchange program between cells and the tissue environment, which would contribute to the neuroprotective effect.

The promotion of OPC generation would contribute and accelerate the re-myelination process after SCI. Diverse clinical trials [45,46] based on previous successful pre-clinical studies [47,48,49,50] have tried to activate oligodendrocyte replacement for inducing re-myelination. FM19G11 was shown before to accelerate the maturation of OPC when the in vitro differentiation process was under the control of the related self-renewal transcriptional machinery in hypoxia [30]. Here, we show that FM19G11 can accelerate the differentiation and maturation of epSPCs in vitro derived from adult spinal cord also under normoxic conditions, showing a relevant effect of this compound in the reprogramming of the undifferentiated progenitors into mature lineages independently in certain micro-environmental conditions. FM19G11 modulates the expression of Sox2, Oct4 and Nanog depending on the oxygen tension to which the precursor cells are exposed [27,30]. Along the oligodendrocyte-directed differentiation process, FM19G11 more quickly decreased Sox2, Oct4 and PCNA undifferentiated and proliferative markers, respectively, in parallel with the increased expression of mature oligodendrocytes like RIP. Interestingly, FM19G11 was able to significantly induce the OPC population, revealed by the increased signal of Olig1, an early oligodendrocyte marker, showing an additive effect on the combinatory treatment with epSPCi. No significant co-localization of epSPCi and Olig1 was found; however, because all samples were studied at the time of sacrifice, two months after injury, a direct influence on the differentiation of the transplanted cells by FM19G11 cannot be discarded.

## 4. Materials and Methods

### 4.1. Ethical Statement Regarding the Use of Animals

Female 2-month-old Sprague Dawley rats (weighing ~200 g) from Charles River and SD-Tg(GFP)2BalRrrc from Rat Resource & Research Center (University of Missouri Columbia, Columbia, MO, USA) were bred at the Animal Experimentation Unit of the Research Institute Príncipe Felipe (Valencia, Spain). The experimental protocol included humanized endpoint criteria by using a score for body weight changes (>20%), body condition (lethargy, pain), autophagy or severe ulcerations and was previously approved by the Animal Care Committee of the Research Institute Principe Felipe (Valencia, Spain) with the protocols identified as 10-0181 and 09-0131 in accordance with the National Guide to the Care and Use of Experimental Animals (Real Decreto 1 December 2005).

### 4.2. EpSPCi Cell Culture

EpSPCi (eGFP+/+ for cell transplantation in vivo or eGFP^−/−^ for in vitro assays) were isolated from adult female Sprague Dawley rats and SD-Tg(GFP)2BalRrrc five days after severe contusion of spinal cord (250 kdyn at T8–T9, see above) and cultured as previously described [25,51].

Oligodendrocyte precursor cell (OPC)-induced differentiation was performed as previously described [25] with slight modifications, including in the FM19G11 condition 500 nM of the compound FM19G11 (refreshed every two days) or the corresponding amount of vehicle, DMSO in the control group. The differentiation procedure was performed until Day 21 on culture in order to induce the early oligodendrocyte precursors. Part of the OPCs was destined to immunostainings for RIP marker and the other part for total mRNA and protein isolation for quantitative PCR of NG2 and Western blotting detection of Oc4, Sox2 and PCNA. Three independent experiments were performed.

### 4.3. Spinal Cord Contusion, epSPCi Transplantation, Intrathecal Drug Administration and Functional Locomotor Analysis

SCI by contusion was performed as previously described [25]. Briefly, severe contusion (250 kdyn, “Infinite Horizon Impactor”) at thoracic segment T8 was performed. For intramedullary transplantation, 10^6^ in 10 μL of total volume epSPCi were transplanted by stereotaxis distributed into rostral and caudal regions at a distance of 2 mm from the lesion at a rate of 2 μL/min by using a 30 G Hamilton pipette filled with cell suspension and mounted on the microinjector. We waited 1 min between injections before moving the syringe to allow cell deposition into the medullar tissue. The compound FM19G11 or DMSO (as vehicle control) was administrated via intrathecal catheterization. Partial laminectomy at T13 allows introducing the catheter (Alzet Corp., Cupertino, CA, USA; previously filled with 0.9% saline solution) through a perforation in dura mater, up to the injured segments (T8). The osmotic pump, Model 1007D (Alzet Corp. Germany; previously filled with FM19G11 or DMSO and incubated overnight at 37 °C in a saline solution), delivers 0.5 μL per hour of a 9 mM FM19G11 solution or DMSO (vehicle) during 3 days.

The rats were pre-medicated with subcutaneous morphine (2.5 mg/kg) and Baytril (enrofloxacin, 5 mg/kg, Bayer, Leverkusen, Germany) and anesthetized with 2% isoflurane in a continuous oxygen flow of 1 L/min. All animals were subjected to post-surgery care and passive and active rehabilitation protocols as previously described [25]. Open-field locomotion was evaluated by two blinded observers by using the 21-point BBB locomotion scale after blind visualization of a minimum of 5 min of free walking in an open space once a week [52]. The animals were sacrificed after 8 weeks of evaluation.

### 4.4. Histology and Quantification of the Tissue Volume at the Injury Area

The animals were transcardially perfused with a 0.9% saline solution followed by 4% PFA in PBS and 2 days incubation time in 30% sucrose before inclusion in Tissue-Teck OCT (Sakura Finetek Barcelona, Spain). Sagittal cryosections of a 10-μm thickness were used for immunoassays. Every fifth section was collected for eosin and hematoxylin (E&H) staining to determine the anatomical structure and tissue volume calculations in the injured area. E&H-stained sections were scanned in a Pannoramic 250 Flash II scanner (3DHISTECH Ltd.; Budapest, Hungary), and images of approximately 20 mm^2^ of medullar tissue (including the epicenter of the lesion) were acquired with the Pannoramic viewer software. The quantification of the spinal cord width was done at eight different points within the injured area. Using Adobe Photoshop^®^ software (version CS2, San Jose, CA, USA), the images of every section, from dorsal to ventral areas, were placed consecutively, and 8 guidelines were traced vertically separated 0.5 mm along the epicenter of the injury. Spinal cord width was measured in every eighth point of every section following the guidelines using the scale bar for absolute quantification and normalization in mm.

The total thickness of the SC at the site of the lesion was quantified by including all the consecutive slides and multiplying them by the thickness of every cryosection (10 μm). Considering the elliptic-shape of the SC, the area quantification was calculated as follows:

Area (mm^2^) = higher radius (higher diameter/2) × smaller radius (smaller diameter/2) × π

### 4.5. Immunocytochemistry and Immunohistochemistry

Cells or cryosectioned tissues (10 μm) were post-fixed with 4% paraformaldehyde at room temperature for 10 min. After permeabilization with PBS containing 0.5% Triton and 2% goat serum (blocking solution), the primary antibodies were incubated overnight at 4 °C. Cells or tissue sections were incubated with GAP43 (α-rabbit; Cat. ab128005 Abcam, Hong Kong, China), β-tubulin III (α-mouse; Cat. MO15052 Neuromics, Edina, MN, USA), GFAP (α-rabbit; Cat. Z0334 DAKO, Santa Clara, CA, USA), RIP (α-mouse; Cat. MAB1580 Chemicon, Pittsburgh, PA, USA) and OLIG1 (α-rabbit; Cat. AB5320 Chemicon). Primary antibodies were diluted 1:200 in blocking solution. After being rinsed three times with PBS, the cells or the tissue sections were incubated with Oregon Green-Alexa488, Alexa555 or Alexa647 dye-conjugated secondary antibodies for 1 h at room temperature. All cells and tissue sections were counterstained by incubation with DAPI for 5 min at room temperature followed by washing steps. Signals were visualized by both fluorescent microscopy (fluorescence microscope Leica DM6000B, Wetzlar, Germany) and confocal microscopy (confocal microscope Leica TCS-SP2-AOBS, Wetzlar, Germany). The quantification of immunostainings was performed using ImageJ software (Image J2, Madison, WI, USA).

### 4.6. Western Blot

epSPCi neurosphere-like cultures at the indicated points of the OPC differentiation process were lysed in 50 mM Tris-HCl, pH 7.5, 150 mM NaCl, 0.02% NaN3, 0.1 SDS, 1% NP40, 1 mM ethylene diamine tetra acetic acid (EDTA), 2 μg/mL leupeptin, 2 μg/mL aprotinin, 1 mM PMSF and 1 × Protease Inhibitor Cocktail (Roche Diagnostics, San Diego, CA, USA), stored on ice for 30 min and boiled for 10 min. Twenty five µg of each protein extract were subjected to sodium dodecyl sulfate (SDS) polyacrylamide gel electrophoresis (PAGE; MiniProtean^®^, Bio-Rad, Hercules, CA, USA) and blotted with antibodies overnight at 4 °C. Signal detection was performed with an enhanced chemiluminescence kit (ECL Plus Western blotting detection reagent from GE Healthcare, Piscataway Township, NJ, USA). Primary antibodies used were Sox2 (α-mouse; Cat. MAB2018. R&D System, Minneapolis, MN, USA), NG2 (α-mouse; Cat. MAB5384. Chemicon), PCNA (α-mouse; Cat. ab29. Abcam) and β-actin for the loading control (α-mouse; Cat. A5441 Sigma, Sant Louis, MO, USA). At least 3 biologically independent replicates were always performed; a representative Western blot is shown.

### 4.7. RNA Isolation and Quantitative RT-PCR

Total RNA of epSPCi at the indicated points of the OPC differentiation process was extracted by using the RNeasy Mini-kit (Qiagen, Hilden, Germany) according to the manufacturer’s instructions. One microgram of total RNA was reverse-transcribed in a total reaction volume of 50 μL at 42 °C for 30 min using random hexamer primers. For quantitative analyses, we used LightCycler SYBR^®^ Green I technology, and the relative expression of mRNA transcripts was analyzed by the ABI PRISM 5700 Sequence Detection System (Applied Biosystems, Foster City, CA, USA). As a template, we used 40 ng of cDNA to analyze the expression of target and housekeeping genes (18S) in separate tubes for each pair of primers. The sequences from 5′ to 3′of each pair of primers are: rNG2, forward primer: 5′-ATGCTTCTCAGCCCGGGACA-3′; reverse primer: 5’-GGTTGCGGCCATTGAGAATG-3’; r18S, forward primer: ggaagggcaccaccaggagt; reverse primer: tgcagccccggacatctaag. The comparative threshold cycle (Ct) method was used to calculate the relative expression as follows:

Δ*C*_t_ (Δ*C*_t_ = *C*_t_ (test gene) − *C*_t_ (GAPDH))

Δ*C*_t_ for FM19G11-treated samples was then subtracted from the Δ*C*_t_ for vehicle-treated samples, to generate ΔΔ*C*_t_ (ΔΔ*C*_t_ = Δ*C*_t_ (*FM19G11*) − Δ*C*_t_ (vehicle)). The mean of these ΔΔ*C*_t_ measurements was used to calculate the fold change in gene expression (2 ^−ΔΔ*C*t^). Results were presented as the mean ± standard error.

### 4.8. Statistical Analysis

Student’s *t*-test was performed for pair comparisons and one-way analysis of variance (ANOVA) for multiple value comparisons. All measurements in cell culture experiments were carried out in at least three different culture preparations, and the results were expressed as the mean ± SEM. Statistical analysis was performed using GraphPad Prism 4 Project software (San Diego, CA, USA). In all cases, *p* < 0.05 was considered significant.

## 5. Conclusions

Altogether, significant beneficial cellular changes at the histological level were found in the combinatory treatment with epSPCi and FM19G11 in the SCI acute rat model. The obtained results justify the combination of both strategies, cell transplantation and pharmacological treatment, as opposed to the use of the individual ones, for better success on locomotor improvements after SCI, although further investigation would be necessary to demonstrate plausible functional benefits by adjusting doses and/or treatment administrations for long-term treatment and analysis.

## Abbreviations

BBB	Basso, Beattie and Bresnahan
BSCB	Blood-spinal cord barrier
DMSO	Dimethyl sulfoxide
epSPC	Ependymal stem/progenitor cells
epSPCi	Ependymal stem/progenitor cells of the spinal cord after injury
GFAP	Glial fibrillary acidic protein
GFP	Green fluorescence protein
H&E	Hematoxylin-eosin
HIF	Hypoxia inducible factor
NPC	Neural precursor cell
OPC	Oligodendrocyte precursor cell
PFA	Paraformaldehyde
PBS	Phosphate buffer solution
RT-PCR	Reverse transcription-polymerase chain reaction
SC	Spinal cord
SCI	Spinal cord injury
UCP	Uncoupling protein
